# Comparative study on *Toxoplasma* infection between Malaysian and Myanmar pregnant women

**DOI:** 10.1186/s13071-014-0564-9

**Published:** 2014-12-12

**Authors:** Hemah Andiappan, Veeranoot Nissapatorn, Nongyao Sawangjaroen, Myat Htut Nyunt, Yee-Ling Lau, Si Lay Khaing, Khin Myo Aye, Nan Cho Nwe Mon, Tian-Chye Tan, Thulasi Kumar, Subashini Onichandran, Noor Azmi bin Mat Adenan

**Affiliations:** Department of Parasitology, Faculty of Medicine, University of Malaya, 50603 Kuala Lumpur, Malaysia; Department of Microbiology, Faculty of Science, Prince of Songkla University, Hat Yai, Songkhla 90112 Thailand; Department of Medical Research (Lower Myanmar), Republic of the Union of Myanmar, Yangon, Myanmar; Department of Obstetrics and Gynecology, Faculty of Medicine, University of Malaya, 50603 Kuala Lumpur, Malaysia

**Keywords:** *Toxoplasma gondii*, Seroprevalence, Risk factors, Avidity, Pregnant women, Malaysia, Myanmar

## Abstract

**Background:**

*Toxoplasma gondii*, an obligate intracellular protozoan parasite, causes a disease called toxoplasmosis which can sometimes be acquired congenitally by a newborn from an infected mother. This study aimed to determine the seroprevalence of *Toxoplasma* infection and its associated risks among 219 and 215 pregnant women from Malaysia and Myanmar, respectively.

**Methods:**

Anti-*Toxoplasma* IgG and IgM antibodies were screened by using standard commercial ELISA kits. The socio-demographic, obstetrics and risk factors associated with *Toxoplasma* infection data were compared between the two countries.

**Results:**

The overall prevalence of *Toxoplasma* infection in Malaysian pregnant women (42.47%; 95% CI = 36.11-49.09) was significantly higher (*p <* 0.05) than Myanmar pregnant women (30.70%; 95% CI = 27.92-37.16). By univariate analysis, this study identified that age group, education, parity, awareness on toxoplasmosis and consumption of undercooked meat were significantly associated (*p <* 0.05) with *Toxoplasma* seropositive Malaysian pregnant women but none of these factors associated with *Toxoplasma* seropositive Myanmar pregnant women. In comparison using univariate analysis between the two countries, it was found that *Toxoplasma* seropositive Malaysian pregnant women was associated with aged 30 years and above, secondary or lower-secondary level of education, the third trimester of pregnancy, having one child or more, lacking awareness of toxoplasmosis, absence of bad obstetrics history, having no history of close contact with cats or soil, living on a farm and also consumption of undercooked meat, unpasterized milk or untreated water. Avidity measurement was used to confirm the stages of *Toxoplasma* infection in pregnant women who were positive for both IgG and IgM antibodies and found all were infected in the past.

**Conclusion:**

From our study, *Toxoplasma* screening and its risk measurement in pregnant women is firmly recommended for monitoring purposes and assisting proper management, including diagnosis and treatment during antenatal period. Also, it is necessary to initiate preventive measures for *Toxoplasma* infection among reproductive-age women in general and seronegative pregnant women in particular. Avidity measurement should be incorporated in *Toxoplasma* routine screening, especially with the availability of a single serum sample to assist in the diagnosis.

**Electronic supplementary material:**

The online version of this article (doi:10.1186/s13071-014-0564-9) contains supplementary material, which is available to authorized users.

## Background

*Toxoplasma gondii* has a great impact on the human health generally, and more serious outcomes occur in immunocompromised and pregnant women specifically [1]. As reported in many previous studies, the infection sources are consumption of contaminated raw meat [2], water [3], fruits [4] and vegetables [5] or having close contacts with felines [6] and exposure to soil contaminated with cats’ feces [7]. Infection during early pregnancy is more serious compared to infection in late gestation, but the likelihood of disease transmission increases during the progression of pregnancy [8]. *Toxoplasma* infection in the first trimester often causes abortion and late infection causes premature birth, or may lead to adverse complications in babies such as enlarged liver and spleen, eye damage from inflammation of the retina or other parts of the eye, jaundice, seizures, mental retardation and even death [9-11]. The detection of anti-*Toxoplasma* antibodies in pregnant women helps in disease management and the proper course of treatment. In recent years, the measurement of IgG avidity had been used to confirm the stages of *Toxoplasma* infection, either recent or past infection in pregnant women. *Toxoplasma* infection is prevalent in most countries and large populations in countries with tropical climates are affected. This study was conducted due to the fact that the seroprevalence of *Toxoplasma* infection in Malaysia pregnant women was recorded a decade ago [12] and no studies have been reported from Myanmar pregnant women [13]. Furthermore, Southeast Asia is a unique region where its local people share similar life styles, traditions and cultures, regardless of their socio-economic backgrounds. This study therefore aimed to demonstrate the current situation concerning the seroprevalence of *Toxoplasma* infection and to identify plausible risk factors among pregnant women from Malaysia and Myanmar. The information obtained could help in better understanding the epidemiological data on *Toxoplasma* infection between these two Southeast Asian nations. Also, it could further strengthen regional collaboration at a larger scale for initially eliminating the infection rate and later eradicating the disease burden of this enigmatic parasite from this region.

## Methods

### Study site and population

**Figure 1:**
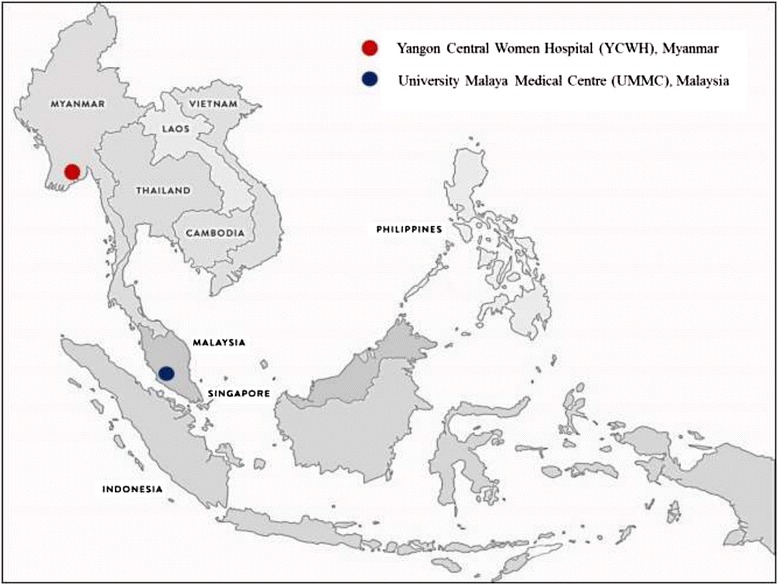
**Study sites in Malaysia and Myanmar.**

This is a prospective cross-sectional study. A total of 219 pregnant women visiting the antenatal clinic (ANC) in University Malaya Medical Centre (UMMC), Malaysia were recruited. UMMC is a government-funded medical institution located in Pantai Dalam, Kuala Lumpur, Malaysia. This hospital was founded in 1962 and has 866 beds. UMMC is a part of the University Malaya, a higher educational institution, and facilitates teaching, research and training for medical personnel. Meanwhile, a total of 215 pregnant women attending ANC at Yangon Central Women Hospital (YCWH), Myanmar were also recruited in this study (Figure 1). YCWH is a public hospital in Yangon, Myanmar, built for women’s health care in the area. This hospital was founded in 1960 and has 750 beds. YCWH provides teaching facilities for the University of Medicine, Yangon, the Yangon Institute of Nursing and the University of Paramedical Science, Yangon. All eligible pregnant women at any gestational age gave informed consent before the commencement of this study.

All the participants’ information related to socio-demographic such as their age, education level, occupation, gestation period, and parity and plausible risk-factors associated with toxoplasmosis (bad obstetrics history, presence of their own cats at home, presence of stray cats at home, consumption of undercooked meat, drinking unpasteurized milk, drinking untreated water, having contact with soil and living on a farm) were recorded in the formatted questionnaire forms. An operational definition was used for the risk factors. Bad obstetrics history was defined as a person having a history of miscarriage, still birth, premature labor, low birth weight, congenital anomalies or prenatal death in their previous pregnancy.

### Ethical consideration

This study was conducted with the approval from the ethical review committee of the University of Malaya Medical Centre, Malaysia (EMC 901.1) and Ministry of Health, Department of Medical Research (Lower Myanmar), Myanmar (ERC Meeting 1/2011), in accordance with the Helsinky Declaration for the inclusion of human subjects in research.

### Serum sample collection

Approximately 5 mL venous blood was drawn from the eligible pregnant women; sera were processed and were kept at −20°C until testing.

### Detection of anti*-Toxoplasma* antibodies

The serum was screened for anti-*Toxoplasma* IgG and IgM antibodies by using standard ELISA commercial kits (IgG-NovaLisa and IgM-NovaLisa, Dietzenbach, Germany) in accordance with the manufacturer’s instruction. A positive sample for both anti-*Toxoplasma* IgG and anti-*Toxoplasma* IgM antibodies was also tested for its avidity using a standard ELISA commercial kit (IgG-NovaLisa Dietzenbach, Germany); high avidity indicates a past infection (of at least 4–5 months) and a low avidity indicates a recently acquired infection (within 4–5 months).

### Statistical analysis

The data collected in the questionnaires and the serology results were analyzed by using statistical software SPSS version 17.0 (SPSS, Inc., Chicago, IL). The qualitative variables were estimated and presented as frequencies and percentages. Univariate analyses and the χ^2^ test were used to investigate the association between *Toxoplasma* seropositivity as a dependent variable and possible demographic and associated factors as independent variables. *p* ≤ 0.05 was regarded as statistical significant.

## Results

### Seroprevalence of *Toxoplasma* seropositivity and IgG avidity measurement

The seroprevalence of *Toxoplasma* infection in Malaysian and Myanmar pregnant women is shown in Table 1. It was found that the seroprevalence of *Toxoplasma* infection in Malaysian pregnant women was significantly higher than in Myanmar pregnant women (*p <* 0.05). Furthermore, IgG avidity of 7 pregnant women (6 Malaysian and 1 Myanmar) who were positive for both the anti-*Toxoplasma* IgG and IgM antibodies indicated past *Toxoplasma* infection.

**Table 1:** **Seroprevalence of** ***Toxoplasma*** **infection in pregnant women from Malaysia and Myanmar as assessed by ELISA**

**Anti-** ***Toxoplasma*** **antibodies**	**Malaysia (N = 219) (n, %)**	**Myanmar (N = 215) (n, %)**	***p*** **-value**
IgG^+ve^	87 (39.73%)	65 (30.23%)	
IgG^+ve^ and IgM^+ve^	6 (2.74%)	1 (0.47%)	
Total	93 (42.47%) (95% CI = 36.11-49.09)	66 (30.70%) (95% CI = 27.92-37.16)	0.011

### The associations between the demographic profiles and associated factors of *Toxoplasma* seropositivity in pregnant women from Malaysia and Myanmar

The age range of the pregnant women from Malaysia was 20 to 41 years with a mean of 30.07 ± 4.43 years while the pregnant women from Myanmar was 18 to 45 years with a mean of 29.46 ± 5.50 years. The majority of the pregnant women from Malaysia had higher education and were employed, but the majority of the pregnant women from Myanmar had only secondary or lower education and was unemployed. Most of the Malaysian pregnant women were in their third trimester and had one or more children but the Myanmar pregnant women were in their third trimester and expecting their first born. The association between *Toxoplasma* seropositive pregnant women and their demographic profiles and risk factors from each country were determined by univariate analysis. It was found that factors such as age group, level of education, parity, awareness on toxoplasmosis and consumption of undercooked meat shown significant associations (*p <* 0.05) with Malaysian pregnant women. Meanwhile, none of these variables played a significant role in Myanmar seropositive pregnant women (Table 2).

**Table 2:** **Univariate analysis on demographic profiles and associated factors of** ***Toxoplasma*** **seropositivity in Malaysian and Myanmar pregnant women**

**Variables**	**Malaysia (N = 219)**			**Myanmar (N = 215)**		
	**Total (%)**	**IgG** ^**+ve**^ **(%)**	***p*** **-value**	**Total (%)**	**IgG** ^**+ve**^ **(%)**	***p*** **-value**
Age group			0.042			0.179
<30	107 (48.9)	38 (35.5)		109 (50.7)	38 (34.9)	
≥30	112 (51.1)	55 (49.1)		106 (49.3)	28 (26.4)	
Education			0.006			0.444
≤secondary	74 (33.8)	41 (55.4)		132 (61.4)	38 (28.8)	
>secondary	145 (66.2)	52 (35.9)		83 (38.6)	28 (33.7)	
Occupation			0.578			0.954
Employed	178 (81.3)	74 (41.6)		69 (32.1)	21 (30.4)	
Unemployed	41 (18.7)	19 (46.3)		146 (67.9)	45 (30.8)	
Gestation			0.684			0.144
1^st^ trimester	5 (2.3)	3 (60.0)		6 (2.8)	4 (66.7)	
2^nd^ trimester	79 (36.1)	32 (40.5)		95 (44.2)	27 (28.4)	
3^rd^ trimester	135 (61.6)	58 (43.0)		114 (53.0)	35 (30.7)	
Parity			0.043			0.277
None	42 (19.2)	12 (28.6)		125 (58.1)	42 (33.6)	
≥1	177 (80.8)	81 (45.8)		90 (41.9)	24 (26.7)	
Awareness on toxoplasmosis			0.005			0.179
Yes	37 (16.9)	8 (21.6)		4 (1.9)	0 (0)	
No	182 (83.1)	85 (46.7)		211 (98.1)	66 (31.3)	
Bad obstetrics history			0.907			0.217
Yes	21 (9.6)	9 (42.9)		13 (6)	2 (15.4)	
No	198 (90.4)	84 (42.4)		202 (94)	64 (31.7)	
Presence of own cats at home			0.489			0.387
Yes	30 (13.7)	11 (36.7)		40 (18.6)	10 (25)	
No	189 (86.3)	82 (43.4)		175 (81.4)	56 (32)	
Presence of stray cats at home			0.100			0.690
Yes	17 (7.8)	4 (23.5)		74 (34.4)	24 (32.4)	
No	202 (92.2)	89 (44.1)		141 (65.6)	42 (29.8)	
Consumption of undercooked meat			0.015			0.175
Yes	22 (10)	4 (18.2)		10 (4.7)	5 (50)	
No	197 (90)	89 (45.2)		205 (95.3)	61 (29.8)	
Drinking unpasteurized milk			*			0.901
Yes	0 (0)	0 (0)		7 (3.3)	2 (28.6)	
No	219 (100)	93 (42.5)		208 (96.8)	64 (30.8)	
Drinking untreated water			*			0.494
Yes	0	0 (0)		113 (52.3)	37 (32.7)	
No	219 (100)	93 (42.5)		102 (47.4)	29 (28.4)	
Contact with soil			0.351			0.366
Yes	32 (14.6)	16 (50)		32 (14.9)	12 (37.5)	
No	187 (85.4)	77 (42.2)		183 (85.1)	54 (29.5)	
Living in the farm			0.243			0.901
Yes	1 (0.46)	1 (100)		7 (3.3)	2 (28.6)	
No	218 (99.5)	92 (42.2)		208 (96.7)	64 (30.8)	

*No variance of data.

### Comparison of the association of demographic profiles and risk factors with *Toxoplasma* seropositive pregnant women between Malaysia and Myanmar

The association between the seropositive pregnant women with their demographic profiles and risk exposures from Malaysia and Myanmar were compared by univariate analysis. Our findings showed that, Malaysian pregnant women were more prone to *Toxoplasma* seropositivity compared to Myanmar pregnant women, particularly pregnant women in the age group of 30 years and above, those who had secondary or lower level of education, were in their third trimester, had one or more than one child, lacked of awareness of toxoplasmosis, had no bad obstetrics history, did not own cats or have stray cats at home, did not consume undercooked meat, did not drink unpasteurized milk or untreated water and had no contact with soil or live on a farm (Table 3).

**Table 3:** **Univariate analysis to compare the association between demographic profiles and risk factors with** ***Toxoplasma*** **seropositivity in Malaysian and Myanmar pregnant women**

**Variables (Malaysian)**	**Adjusted odd ratio (95% CI)**
Age ≥30 years	2.69 (1.52-4.75)
Having ≤ secondary level of education	3.07 (1.70-5.56)
In 3^rd^ trimester	1.70 (1.01-2.87)
Having ≥ 1 children	2.32 (1.33-4.03)
No awareness on toxoplasmosis	1.93 (1.28-2.91)
No bad obstetrics history	1.59 (1.06-2.40)
Absence of own cats at home	1.63 (1.06-2.50)
Absence of stray cats at home	1.86 (1.18-2.93)
No consumption of undercooked meat	1.95 (1.29-2.93)
No drinking unpasteurized milk	1.66 (1.12-2.47)
No drinking untreated water	1.86 (1.12-3.08)
No contact with soil	1.67 (1.09-2.57)
No living in Farm	1.64 (1.10-2.45)

## Discussion

*Toxoplasma gondii* causes severe impairment and even death to fetuses or to newborns through congenital infection. In this study, the seroprevalence of *Toxoplasma* infection among pregnant women was compared between two Southeast Asian countries, Malaysia and Myanmar. The statistic analysis indicated that pregnant women from Malaysia have significantly higher *Toxoplasma* seropositive rate when compared to pregnant women from Myanmar (*p <* 0.05). *Toxoplasma* seropositive in Malaysia pregnant women from this study (42.49%) was similar to the previous study (49%), conducted a decade ago [12]. Globally, reported prevalence of *Toxoplasma* infection varies, for example;, 48.6% in Albania [14], 68.6% in Brazil [15], 10.6% in China [16], 75% in India [17], 10.3% in Japan [18], 17.0% in Korea [19], 18.5% in Netherlands [20], 40.8% in Nigeria [21] and 38% in Saudi Arabia [22]. *Toxoplasma* seropositivity in Malaysia were found to be higher than neighboring countries; being 28.3% in Thailand [23], 17.2% in Singapore [24] and 7.7% in Vietnam [25]. Meanwhile, data of *Toxoplasma* infection in Myanmar is scanty as there was only one reported study of school children since 1977 [13] and no work has been published since. This is the first study conducted among pregnant women in Myanmar. The high *Toxoplasma* seropositive in both countries showed the need for routine screening for toxoplasmosis among women of child-bearing age, and pregnant women in particular, for monitoring and preventive purposes.

*Toxoplasma* seropositivity in Malaysian pregnant women showed significant association with the demographic and potential risk exposure factors. The possibility of acquiring *Toxoplasma* infection was high in pregnant women in the age group of 30 years and above, with secondary or lower level of education and who had more than one child. A previous study also shown that prevalence of *Toxoplasma* infection increases by age [26], is associated with a low level of education [27] and having had more than one child [28]. Malaysian pregnant women with a lack of awareness on toxoplasmosis were more prone to acquire *Toxoplasma* infection. Knowledge and awareness about toxoplasmosis, as stated in many studies, may assist these pregnant women in protecting themselves from this parasitic infection [29-31]. As shown in many previous studies [32-34] consumption of undercooked meat (animal tissues may be contaminated by *T. gondii* cysts) is an important mode of disease transmission. Interestingly, our study showed that those who do not consume undercooked meat have higher *Toxoplasma* seropositivity. Based on this finding, it is suggested that further investigations are conducted before any conclusion could be made. Meanwhile, it is a different scenario with the Myanmar pregnant women. There was no statistically significant association found between the Myanmar seropositive pregnant women with the demographic profiles or risk exposures. Future studies with larger sample size and consideration of other confounding factors are needed to explain the acquisition of *Toxoplasma* infection in these Myanmar pregnant women.

The comparison between Malaysian and Myanmar seropositive pregnant women showed that Malaysian pregnant women were more prone to acquire *Toxoplasma* infection than Myanmar pregnant women. Besides the factors such as age group, level of education, parity and consumption of undercooked meat, the awareness on toxoplasmosis showed a significant association with *Toxoplasma* seropositivity in the comparison among the pregnant women between these two countries. Lack of awareness of toxoplasmosis might be due to lack of access to information related to *Toxoplasma* infection, especially amongst pregnant women. As *T. gondii* is a TORCH [acronym for a group of five infectious diseases namely; Toxoplasmosis, Others (Hepatitis B), Rubella (German measles), Cytomegalovirus (CMV), Herpes Simplex Virus (HSV)] infection, women in general, and pregnant women in particular, need to be educated about toxoplasmosis as prevention and control measures [29,35,36]. A total of approximately 1.2 million metric tons of cat feces were being deposited in the environment annually and the annual oocysts burden measured in a community survey was 3 to 434 oocysts per square foot, as reported in a study conducted in USA [37]. Exposure to the contaminated cat, its feces or contaminated soils may pose a risk to pregnant women. However, these Malaysian pregnant women had no contacts with cats (their own or stray cats), soils or did not lives on a farm. Meanwhile, many studies are being conducted on waterborne toxoplasmosis in recent years [38-41], highlighting the possibility of *T. gondii* transmission by the usage of oocysts contaminated water, especially for drinking purpose, but these reports contradict our finding on high *Toxoplasma* seropositivity in Malaysian pregnant women drinking treated water. There could be other confounding factors leading to the findings in this study and there is a need for further investigations. Meanwhile, these pregnant women should be educated [42] in order to prevent and control the acquisition of *Toxoplasma* infection by themselves and their fetus.

All the Malaysian and Myanmar seropositive pregnant women acquired chronic *Toxoplasma* infection, supported by absence of clinical presentation in the fetus, false positive IgM results and confirmed by IgG avidity measurement. Avidity test proves to be a complementary tool in the diagnosis of toxoplasmosis among pregnant women. The persistence of IgM antibodies in the blood stream for many months up to a year, may lead to misdiagnosis of being primary acquired *Toxoplasma* infection, especially in the pregnant woman and her fetus [43]. Measurement of IgG avidity should be included in serological diagnosis, especially with a single serum sample of pregnant women for their first visit to the antenatal clinic to determine the staging of *Toxoplasma* infection consistent with clinical presentation.

## Conclusion

The seroprevalence of *Toxoplasma* infection remains high in both Malaysia and Myanmar. The identified risks of exposure were found to be only associated with *Toxoplasma* seropositive pregnant women in Malaysia. Lack of awareness of toxoplasmosis was one of the factors contributing to high prevalence in these pregnant women from both countries. Hence, a routine screening for toxoplasmosis should be recommended for all women of reproductive age and pregnant women in early pregnancy for monitoring and preventive measures. Besides that, health education and related knowledge of risk exposure should be provided to the general population and pregnant women attending ante-natal clinics, in order to propagate awareness about *Toxoplasma* infection and to reduce the infection rate and its disease burden (multilingual brochures are attached as Additional file 1). Furthermore, IgG avidity measurement provides a useful tool for determining the staging of *Toxoplasma* infection and to assist in the course of treatment among pregnant women in general and in their early pregnancy in particular.

## Additional file

Additional file 1:
**Health care education on toxoplasmosis in pregnant women.**
