# Supplementary material for: Comparative study on Toxoplasma infection between Malaysian and Myanmar pregnant women
Source: Parasit Vectors. 2014 Dec 12;7:564. doi: 10.1186/s13071-014-0564-9 (PMC4297455; doi:10.1186/s13071-014-0564-9)
Supplement: Additional file 1: — Health care education on toxoplasmosis in pregnant women. [file 13071_2014_564_MOESM1_ESM.doc]

**Additional file 1: Health care education on toxoplasmosis in pregnant women.**

Brochures in English Language.

Brochures in Malay Language

Brochures in Chinese Language

Brochures in Tamil (Indian) Language

Brochures in Myanmar Language
